# Advanced Resuscitation with an Intact Cord in Preterm Lambs: A Feasibility Trial

**DOI:** 10.3390/children13050651

**Published:** 2026-05-06

**Authors:** Lida Zeinali, Evan Giusto, Heather Knych, Amy Lesneski, Morgan Hardie, Houssam Joudi, Deepika Sankaran, Satyan Lakshminrusimha, Payam Vali

**Affiliations:** 1 Valley Children’s Medical Group, Madera, CA 93636, USA; 2 Division of Neonatology, Department of Pediatrics, University of California Davis, Sacramento, CA 95817, USA; 3 K.L. Maddy Equine Analytical Pharmacology Laboratory, Department of Veterinary Molecular Biosciences, School of Veterinary Medicine, University of California Davis, Davis, CA 95616, USA; 4 Department of Stem Cell Research, University of California Davis, Sacramento, CA 95817, USA; 5 School of Veterinary Medicine, University of Pennsylvania, Philadelphia, PA 19104, USA

**Keywords:** resuscitation, preterm, epinephrine, delayed cord clamping, chest compressions

## Abstract

**Highlights:**

**What are the main findings?**

- Direct umbilical venous epinephrine administration during delayed cord clamping significantly shortened the time to first epinephrine compared to immediate cord clamping with umbilical vein catheter (UVC) placement.
- Resuscitation with an intact cord achieved similar return of spontaneous circulation rates and epinephrine plasma concentrations compared to immediate cord clamping and UVC epinephrine.

**What is the implication of the main finding?**

- Advanced resuscitation with an intact cord using direct umbilical venous epinephrine is feasible and may allow earlier pharmacologic intervention without delaying stabilization.
- This approach has the potential to minimize chest compressions and improve hemodynamic stability, supporting further investigation as an alternative resuscitation strategy in preterm infants.

**Abstract:**

**Background:** Preterm infants with bradycardia at birth often undergo immediate cord clamping (ICC) followed by resuscitation with positive pressure ventilation (PPV), chest compressions (CCs) and umbilical venous catheter (UVC) epinephrine. Resuscitation with an intact cord (PPV during delayed umbilical cord clamping—DCC) stabilizes cardiac output but delays UVC placement. **Objective:** To evaluate the feasibility of direct epinephrine injection into the umbilical vein during DCC (DCC + direct-epinephrine) compared with ICC and epinephrine administered through a UVC (ICC + cath-epinephrine), and to explore differences in return of spontaneous circulation (ROSC) and need for CCs between these approaches. **Methods:** Eleven preterm lambs (125–127 d gestation) were asphyxiated by cord compression to decrease heart rate (HR) to <30/min. In the ICC + cath-epinephrine group, the cord was immediately cut, lambs received PPV followed by CCs if HR < 60/min, and epinephrine was administered after UVC placement. In the DCC + direct-epinephrine group, cord compression was released when HR < 30/min and PPV was initiated. If HR remained <60/min, epinephrine was injected into the UV using a 25G needle. If ROSC was achieved, DCC was continued for 2 min. If HR < 100/min, the cord was cut and resuscitation was continued as outlined above. Plasma epinephrine concentrations were analyzed. **Results:** All lambs required epinephrine. Time to epinephrine was shorter with DCC + direct-epinephrine, 1.0 (0.7, 1.6) vs. 3.7 min (3.2, 5.2). Fewer lambs with DCC + direct-epinephrine needed CC (2/6 vs. 5/5, *p* = 0.06). ROSC success and plasma epinephrine concentrations were similar. Post-ROSC, heart rates and mean blood pressures tended to be higher in the ICC + cath-epinephrine group. **Conclusions:** In this perinatal lamb model of asphyxial bradycardia, resuscitation with an intact cord with direct umbilical venous epinephrine injection is feasible. Larger studies are required to determine whether this approach reduces the need for CC or improves clinically meaningful outcomes.

## 1. Introduction

Every year, over 60,000 live births in the United States are very preterm (less than 32 weeks of completed gestation) [1]. Delivery room (DR) cardiopulmonary resuscitation, defined by chest compressions with or without epinephrine (EPI) administration in premature infants, has been shown to be associated with higher mortality and worse short and long-term outcomes compared to premature infants who do not require these interventions [2,3,4,5]. The current recommendation for umbilical cord management of infants who are asphyxiated and need resuscitation at birth is to immediately clamp the umbilical cord owing to insufficient evidence to support delayed cord clamping (DCC) in the presence of perinatal distress followed by positive pressure ventilation (PPV) [6,7,8,9]. Recent evidence suggests that initiation of ventilation with an intact umbilical cord (while in placental circulation, i.e., “physiological based cord clamping”) confers an improved physiologic transition at birth [9,10], and clinical trials have demonstrated that newborns in need of resuscitation can be ventilated during DCC [11,12,13,14,15]. The feasibility, benefits and risks of more advanced resuscitation with chest compressions and EPI with an intact cord are not known.

Recent data from perinatal asphyxiated severely bradycardic lambs (heart rate < 60 beats per minute [bpm]) have shown that chest compressions overlying intrinsic heartbeats may compromise carotid and coronary blood flow [16]. In severely bradycardic neonates, pulmonary blood flow may be decreased to the extent that insufficient blood reaches the lungs for adequate gas exchange, thus making ventilation less effective. Since intravenous access is not readily available following birth, the NRP guidelines recommend immediate cord clamping (ICC) followed by resuscitation with PPV and initiating chest compressions if the heart rate remains below 60 bpm to increase cardiac output, followed by EPI (ICC à PPV à compressions à EPI).

DCC has several hemodynamic benefits in preterm infants. Clinical studies [11,12,13,14,15] have demonstrated that resuscitation with an intact cord is feasible, and we hypothesize that administration of EPI directly into the umbilical vein (UV) in an asphyxiated fetal lamb model increases heart rate and cardiac output, improves response to PPV, decreases the need for chest compressions and enables DCC (PPV à EPI à DCC à compressions). We hypothesize that direct EPI injection into the UV during DCC (DCC + direct-EPI) has similar success and quicker time to return of spontaneous circulation (ROSC) compared to immediate cord clamping (ICC) and EPI administered through a UV catheter (ICC + cath-EPI). We also hypothesize that lambs randomized to DCC + direct-EPI have a decreased probability of requiring chest compressions compared to the ICC + cath-EPI group.

## 2. Materials and Methods

### 2.1. Animal Preparation

The protocol was approved by the Institutional Animal Care and Use Committee (IACUC) at the University of California, Davis (protocol #22544). All experiments were performed in compliance with the ARRIVE guidelines [17]. Experiments were conducted as previously described [18]. Time-dated pregnant ewes (125–127 days gestation, term ~145 days; Dorper-cross) were procured from Van Laningham Farm, Arbuckle, CA, USA. Following an overnight fast, the ewe was sedated with intravenous diazepam and ketamine and intubated with a 9.5 mm cuffed endotracheal tube (ETT). Generalized anesthesia was provided with 2–3% inhaled isoflurane. The ewe was continuously monitored with a pulse oximeter and an end-tidal CO_2_ monitor. Following a cesarean section, the fetal lamb was partially exteriorized to expose the head, neck and right forelimb for pulse oximeter (SpO_2_) probe placement. The lambs were intubated with a 3.5 mm cuffed ETT, and the lung liquid was passively drained by gravity. A catheter was placed in the right carotid artery to measure invasive blood pressures, monitor preductal arterial blood gases and collect samples for plasma EPI concentrations. The right jugular vein was catheterized for fluid and medication administration. A 2 or 3 mm ultrasonic flow probe was placed around the left carotid artery for blood flow measurements.

### 2.2. Experimental Protocol

**Figure 1:**
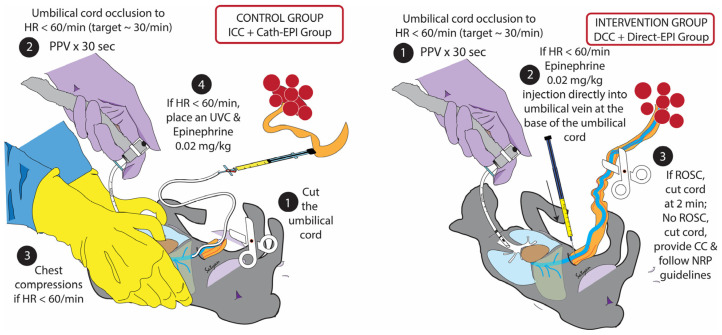
Methods. All lambs underwent umbilical cord compression to induce bradycardia. The control group included 5 lambs with immediate cord clamping (ICC) followed by positive pressure ventilation (PPV), chest compressions and umbilical venous catheter (UVC) placement with epinephrine administration. The intervention group included 6 lambs with delayed cord clamping (DCC) and PPV with an intact cord, followed by direct injection of epinephrine into the umbilical vein with a 25G needle.

Eleven lambs were randomized into two groups (Figure 1) using an opaque sealed envelope.

Following instrumentation, the lamb was exteriorized, placed in a plastic bag, and the umbilical cord was pinched to induce asphyxia and fetal bradycardia. When the heart rate dropped to 30 bpm, the following occurred:

(1) Control group (ICC + cath-EPI): The umbilical cord was immediately tied, the lamb delivered and placed on the radiant warmer. Positive pressure ventilation (PPV) was initiated with a T-piece resuscitator at 35/5 cm H_2_O and 30% O_2_. If the heart rate remained below 60 bpm after 30 s of effective ventilation, chest compressions were started at a compression-to-ventilation ratio (C:V) of 3:1 and oxygen increased to 100%. Preparations to place a UV catheter (UVC) began upon initiation of chest compressions. Once the UVC was inserted and if the heart rate remained <60 bpm, EPI at 0.02 mg/kg (followed by a flush of 3 mL of normal saline) was administered. EPI was repeated every three minutes until ROSC or a maximum of 4 doses.
(2) Intervention group (DCC + direct-EPI): The pinch to compress the umbilical cord was released, and the lamb was ventilated as described above with an intact cord. If the heart rate remained <60 bpm, EPI at 0.02 mg/kg (reconstituted in 3 mL of normal saline) was directly injected into the UV at the base of the umbilicus utilizing a 25-gauge butterfly needle. If the heart rate increased over 100 bpm, DCC was continued for a total of two minutes. If the heart rate remained between 60 and 100 bpm, the umbilical cord was clamped and cut at one minute, and the lamb was delivered onto the radiant warmer. If the heart rate remained <60 bpm, chest compressions were started, and after one minute, the umbilical cord was clamped and cut, and the lamb was delivered. Resuscitation continued per NRP guidelines with chest compressions provided in a 3:1 ratio to ventilation. Preparations were made to place a UVC, and subsequent EPI was administered through the UVC. EPI was repeated every three minutes until ROSC or a maximum of 4 doses.

ROSC was defined as a heart rate >100 bpm and a mean blood pressure >30 mm Hg. Hemodynamic parameters (blood pressure, carotid blood flow and heart rate) were continuously measured and recorded using data acquisition software at a 2000 Hz sample rate (BIOPAC systems MP200, Goleta, CA, USA). Blood was collected at fetal baseline, asystole, one minute before and one minute after each EPI administration, at the time of ROSC, and at one, five, ten and fifteen minutes after ROSC for arterial and venous blood gas analysis as well as plasma EPI concentration. EPI plasma concentrations were analyzed by liquid chromatography-mass spectrometry.

### 2.3. Euthanasia

All animals were euthanized using appropriate methods as described in the NIH Guidelines for the Care and Use of Laboratory Animals. Ewes received an overdose of IV sodium pentobarbital (100 mg/kg), followed by bilateral thoracotomy as a secondary measure to ensure death. Newborn lambs were sacrificed with an overdose of pentobarbital (100 mg/kg IV). These procedures were carried out as recommended by the panel on euthanasia of the American Veterinary Medical Association.

### 2.4. Statistical Analysis

As a feasibility study, we intended to study 10 lambs. Categorical variables were analyzed using the χ^2^ test with Fisher’s exact test as required. Continuous variables are reported as median (IQR) and were analyzed using the Mann–Whitney U test. SPSS 24 (IBM, Armonk, NY, USA) was used for statistical analysis. Nonlinear regression analysis was performed on plasma EPI concentration versus time data, and pharmacokinetic parameters were estimated with compartmental analysis and a user-defined model using Phoenix WinNonlin 64 v8.1 (Princeton, NJ, USA). Goodness of fit and the appropriate weighting factor were selected based on visual analysis of observed versus predicted concentration graphs and residual plots, as well as coefficient of variation, Akaike information criterion (AIC) [19] and Schwarz’s Bayesian Criteria (SBC) [20]. Statistical significance was defined as *p* < 0.05.

## 3. Results

All 11 lambs in this study were profoundly bradycardic following cord compression, and the heart rate did not significantly improve with PPV alone. In the control group, all lambs required chest compressions followed by EPI through the UVC. In the intervention group, all lambs received EPI. Four lambs (67%) had an increase in heart rate >100/min, and DCC was performed at 2 min. One lamb became bradycardic after delivery and required chest compressions, leading to ROSC. The other lamb remained bradycardic in spite of chest compressions and needed UVC placement (which was delayed due to technical difficulties) and received a late dose of UVC EPI to achieve ROSC at 13.9 min.

### 3.1. Return of Spontaneous Circulation and Need for Chest Compressions

Lamb characteristics and arterial blood gases at baseline and at the time of asphyxia were similar between groups (Table 1). ROSC success was similar between groups (4/5 lambs in the control and 6/6 lambs in the intervention group). The time to first EPI administration was shorter in the intervention group (DCC + direct-EPI) with a median (IQR) time of 1.0 (0.7, 1.6) min versus 3.7 (3.2, 5.2) min in the control group (*p* < 0.01; Table 1).

The time to ROSC was not statistically different. However, the time to ROSC in the intervention group was skewed by one lamb who achieved ROSC at 13.9 min because of difficulties in placing the UVC. As a post hoc descriptive analysis, when this lamb was removed from the analysis, the median (IQR) time to ROSC in the intervention group (2 [1.5, 2.7] min) was significantly shorter than in the control group (5.7 [5.2, 5.8] min; *p* = < 0.01; see Supplemental Table S1). Fewer lambs in the intervention group required chest compressions, although this difference did not reach statistical significance (2/6 vs. 5/5, *p* = 0.06). None of the lambs achieved ROSC without EPI, and one lamb in each group required two doses of EPI. All lambs that achieved ROSC survived for one hour, at which time they were euthanized. The one lamb in the control group that did not achieve ROSC received the maximum of four doses of EPI without successful resuscitation and was euthanized per protocol.

### 3.2. EPI Plasma Concentrations

**Figure 2:**
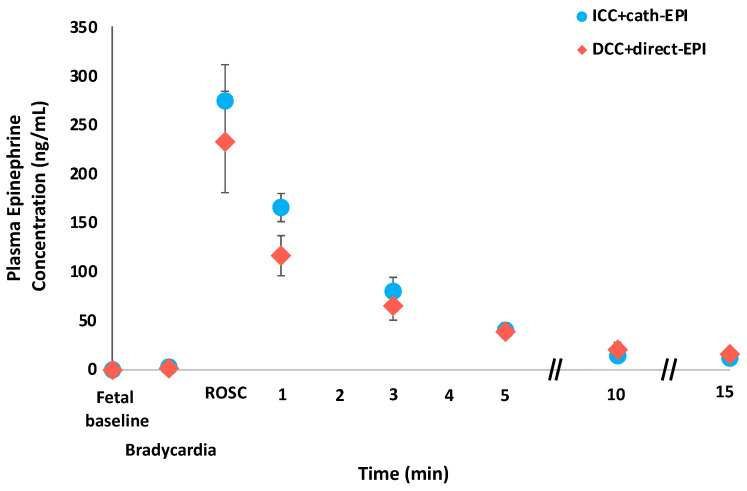
Plasma epinephrine concentrations. Peak epinephrine concentrations were achieved at the time of return of spontaneous circulation (ROSC). Concentrations were similar between groups and steadily declined post-ROSC. Values represented as mean (±SEM). // indicates a break in the linearity of the x time axis.

The mean (±SEM) EPI plasma concentrations in the intervention group were similar to EPI administered after delivery through a UVC, with peak concentrations reached at the time of ROSC, of 233 (±52) ng/mL vs. 275 (±37) ng/mL, respectively (Figure 2). There was a steady decline in plasma EPI concentrations with mean values < 20 ng/mL by 15 min post-ROSC.

### 3.3. Hemodynamic Parameters

Values for heart rate, arterial blood pressure, left carotid blood flow (Q_CA_), and oxygen delivery (DO_2_) to the brain between groups are shown in Table 2. Median (IQR) heart rate was significantly higher in the control group, 183 (174, 191) bpm, compared to the intervention group, 115 (102, 137) bpm, at the time of ROSC. Median (IQR) blood pressure at time of ROSC was higher in the intervention group, 63 (56, 64) mm Hg, vs. 36 (30, 38) mm Hg in the control group. However, in the post-ROSC period, median blood pressure was significantly higher in the control group (ranging 61–68 mm Hg) compared to the intervention group (ranging 46–59 mm Hg). There was no difference in left Q_CA_ and cerebral DO_2_ throughout the study period.

### 3.4. Hematologic Parameters

The hemoglobin at baseline was similar between the two groups: 12.3 (12.1, 12.3) g/dL in the control group and 14.7 (11.6, 15.4) g/dL in the intervention group (*p* = 0.54). Fifteen minutes after ROSC, the hemoglobin concentrations remained similar between the two groups: 11.8 (9.3, 13.1) vs. 12.1 (11.5, 12.2), respectively (*p* = 0.92).

### 3.5. Secondary Analysis

To accurately assess the benefits of two minutes of DCC during advanced resuscitation, we compared the five lambs in the control group to the four lambs in the intervention group whose heart rate increased >100 bpm with EPI and had DCC (Supplemental Tables S1 and S2).

## 4. Discussion

In this study, we have demonstrated that advanced resuscitation with an intact cord is feasible in preterm lambs. This is the first study to assess EPI pharmacokinetics in a premature fetal lamb model that closely mimics the human fetus, as well as to evaluate the feasibility of administering EPI during intact placental circulation. We have shown that asphyxiated, severely bradycardic, premature lambs can be successfully resuscitated with direct injection of EPI into the base of the UV during DCC.

Studies in anesthetized preterm lambs have shown that DCC until ventilation is established (“physiology-based cord clamping”) improves cardiovascular function by increasing pulmonary blood flow, which results in stable cardiac output, and therefore a smoother cardiovascular and cerebral hemodynamic transition [10]. Initiation of assisted ventilation during DCC enhances pulmonary venous return to supplement umbilical venous return to stabilize left ventricular preload and cerebral perfusion [9,11,21]. We have shown that the majority (four out of six) lambs in our intervention group had an increase in heart rate above 100 bpm following EPI administration, which enabled continuing DCC prior to cutting the umbilical cord and delivering the lamb.

A recent review of the use of chest compressions for persistent neonatal bradycardia in the delivery room contends that there is a lack of clear evidence for the use of chest compressions in bradycardic neonates [16]. Neonatal lamb data examining the effects of bradycardia on coronary, carotid and pulmonary blood flows during asphyxia-induced-bradycardia in a transitional circulation model with an open ductus found that unless the heart rate is extremely low or zero, compensatory cardiac mechanisms appear to maintain cerebral perfusion and coronary pressure [16]. The authors speculate that the asynchrony of chest compressions in a heart with a perfusing rhythm (i.e., bradycardia) could play a role in aggravating ischemia, leading to pulselessness and cardiac arrest during resuscitation. In our study, four out of six lambs in the intervention group achieved ROSC without the need for chest compressions, whereas all lambs in the control group received chest compressions. The optimal order of resuscitation steps—PPV à compressions à EPI vs. PPV à EPI à compressions requires further study.

Lambs that received DCC + direct-EPI also achieved a higher mean arterial pressure at the time of ROSC compared to the ICC + cath-EPI lambs, and tended to have lower mean arterial pressure and lower heart rate in the post-resuscitative period. Although abrupt elevations in blood pressure following resuscitation have been associated with cerebral injury, the absolute differences observed in our study were modest, variability was high, and the clinical significance of these post-resuscitation hemodynamic differences is uncertain. We speculate that EPI injection with an intact cord results in greater volume of distribution in the feto-placental unit, which may produce slightly lower EPI levels and a more gradual hemodynamic response. Higher left ventricular preload during DCC may also contribute to the lower heart rate and better blood pressure at ROSC in the intervention-group lambs. Similar lower heart rates at 1 and 5 min with DCC have been reported in clinical trials evaluating resuscitation of non-breathing neonates [22].

Katheria et al. conducted a randomized controlled trial of at-risk vaginally delivered neonates ≥ 37 weeks’ gestational age [11]. Neonates were randomized to receive 1 min or 5 min DCC. Resuscitation included stimulation, blow-by oxygen, and positive pressure ventilation by endotracheal tube or mask. They demonstrated that 5 min DCC was feasible and could be accomplished safely without compromising the ability to perform resuscitation and was associated with increased cerebral tissue oxygen saturation, decreased fractional cerebral tissue oxygen extraction, and greater blood pressure measurements at 12 h of life. There was also a trend for less resuscitation and improved Apgar scores in the 5 min DCC group. In the Baby-Directed Umbilical Cord Clamping (Baby-DUCC) trial led by Blank et al., the umbilical cord remains intact until the infant’s lungs are exchanging gases [12]. They found that it is feasible to provide resuscitation to infants ≥32 weeks’ gestation during DCC, after both vaginal and cesarean births, clamping the umbilical cord only when the infant is physiologically ready. Current evidence suggests that bedside resuscitation with the umbilical cord intact is feasible, safe, and effective [13,14,23,24].

Finally, our EPI pharmacokinetic data confirm that EPI effectively reaches the fetal circulation when administered directly into the UV during DCC. Interestingly, the EPI plasma concentrations were similar between groups. These results suggest that most of the EPI in the intervention group remained within the fetal circulation, although a slightly lower level could be attributed to a larger volume of distribution in the placenta. Previous published data in terms of asphyxiated lambs that received one dose of EPI at 0.03 mg/kg yielded comparable plasma concentrations approximating 400–500 ng/mL [25].

While our findings support the technical feasibility of direct UV EPI injection in this experimental model, several considerations warrant further investigation before clinical translation. The apparent benefit observed in the intervention group, a shorter time to first EPI dose, fewer chest compressions, and more stable post-ROSC hemodynamics, may be driven by earlier access rather than by superior pharmacologic or physiologic effect of the route itself. Direct injection into the UV with a needle in the delivery room raises potential safety concerns, including bleeding from the puncture site, vascular injury, or infection. Operator training, sterile technique, and the practical logistics of performing direct UV injection during ongoing resuscitation in the human delivery room will need to be carefully considered in the design of future clinical studies.

There are several limitations to this study. The sample size was small, as this was a feasibility study, and findings should therefore be regarded as exploratory and preliminary rather than as confirmatory evidence of effectiveness. With a small sample size, individual technical events, such as the delay in placing a UVC in one intervention lamb, can substantially influence results. The post hoc analysis of time to ROSC, excluding this lamb, is descriptive and is reported only to characterize the influence of this single event and should not be interpreted as a confirmatory finding. The study needs to be repeated with a larger sample size, informed by the preliminary data from this study. We did not study an arm with chest compressions followed by EPI with an intact cord, nor did we evaluate whether this approach is effective in asystole without intrinsic heartbeats. The acute experimental design did not permit assessment of safest endpoints related to direct UV puncture or longer-term neurologic outcomes. We intend to conduct additional studies to address these limitations.

## 5. Conclusions

In the severely bradycardic perinatal asphyxiated preterm lamb model, advanced resuscitation with an intact cord by injecting EPI directly into the umbilical vein during DCC is feasible and was associated with a shorter time to first EPI. Whether this approach reduces the need for chest compressions or improves clinically meaningful outcomes requires confirmation in adequately powered studies. Larger studies evaluating direct umbilical venous EPI during DCC and administering EPI prior to chest compressions are needed before consideration for clinical trials.

## Figures and Tables

**Table 1:** Lamb characteristics and resuscitation data.

Groups	Control (*n* = 5)	Intervention (*n* = 6)
Weight (kg)	3.5 (3.0, 3.6)	2.79 (2.3, 3.0)
Gestation (days)	126 (125, 126)	125 (125, 125)
Sex (F:M)	2:3	4:2
Arterial pH		
Baseline	7.21 (7.21, 7.23)	7.23 (7.22, 7.26)
Asphyxia	6.87 (6.84, 6.94)	6.86 (6.85, 6.90)
ROSC	6.78 (6.76, 6.80)	6.82 (6.79, 6.84)
5 min post ROSC	6.71 (6.69, 6.73)	6.77 (6.74, 6.77) *
15 min post ROSC	6.75 (6.74, 6.76)	6.74 (6.73, 6.85)
Arterial PO_2_ (mm Hg)		
Baseline	23 (18, 24)	21 (18, 26)
ROSC	18 (17, 23)	14 (10, 28)
5 min post ROSC	33 (32, 34)	20 (16, 26)
15 min post ROSC	68 (54, 88)	62 (39, 86)
Arterial PCO_2_ (mm Hg)		
Baseline	71 (70, 76)	63 (61, 68)
Asphyxia (mm Hg)	136 (120, 136)	137 (126, 138)
ROSC (mm Hg)	139 (132, 146)	139 (115, 139)
5 min post ROSC (mm Hg)	158 (152, 165)	131 (118, 144) *
15 min post ROSC (mm Hg)	137 (136, 142)	127 (122, 142)
Arterial Lactate (mmol/L)		
Baseline	2.6 (2.1, 3.2)	2.5 (1.7, 3.6)
Asphyxia	7.3 (7, 7.5)	8.3 (7.3, 8.6)
ROSC	9.5 (9.4, 9.9)	9.0 (8.3, 9.9)
5 min post ROSC	8.7 (8.5, 9.6)	9.3 (8.7, 10)
15 min post ROSC	8.3 (8.0, 9.3)	8.9 (8.1, 9.9)
FIO_2_		
At ROSC	1 (1, 1)	0.60 (0.6, 0.9) *
5 min post ROSC	0.85 (0.6, 1)	1 (1, 1)
15 min post ROSC	0.60 (0.5, 0.7)	1 (0.8, 1)
Time to HR <30/min (min)	18 (11.4, 22.2)	22 (14.6, 24.6)
Time to first EPI (min)	3.7 (3.4, 5.2)	1.0 (0.7, 1.6) *
Got chest compressions (%)	5 (100%)	2 (33%)
ROSC success	4 (80%)	6 (100%)
>one EPI dose	1 (20%)	1 (17%)
Time to ROSC (min)	5.7 (4.4, 5.7)	2.4 (1.6, 3.9)

Values represented as median (IQR). HR = heart rate; ROSC = return of spontaneous circulation. * *p*-value < 0.05.

**Table 2:** Hemodynamic data.

Time	Heart Rate (bpm)		Mean BP (mmHg)		Left Q_CA_ (ml/kg/min)		DO_2_ (ml O_2_/kg/min)	
	Control	Intervention	Control	Intervention	Control	Intervention	Control	Intervention
Fetal baseline	133 (115, 163)	143 (139, 154)	45 (42, 50)	49 (43, 52)	34 (30, 34)	34 (30, 38)	3.3 (2.9, 3.5)	3.3 (2.9, 4.9)
Bradycardia	45 (41, 47)	46 (44, 51)	5 (4, 14)	18 (17, 21)	1 (1, 3)	2 (2, 2)	0.0 (0, 0)	0.0 (0, 0)
ROSC	183 (174, 191)	115 (102, 137) *	36 (30, 38)	63 (56, 64) *	13 (9, 20)	26 (16, 34)	0.4 (0.4, 1.5)	0.5 (0.2, 1.5)
1 min post-ROSC	194 (189, 203)	154 (147, 170) *	65 (56, 66)	58 (56, 72) *	18 (16, 24)	34 (25, 42)	0.9 (0.6, 1.1)	0.4 (0.3, 1.6)
2 min post-ROSC	186 (179, 196)	163 (158, 166)	64 57, 67)	59 (50, 67)	22 (15, 25)	28 (20, 40)	1.2 (0.9, 1.9)	0.5 (0.3, 1.2)
3 min post-ROSC	178 (174, 185)	153 (136, 170) *	67 (64, 70)	53 (49, 61) *	23 (14, 24)	29 (19, 39)	1.4 (1.0, 2.1)	1.1 (0.4, 2.3)
4 min post-ROSC	172 (172, 173)	164 (145, 171)	64 (62, 67)	51 (49, 58) *	20 (14, 25)	31 (21, 42)	1.5 (1.2, 1.9)	0.6 (0.7, 1.9)
5 min post-ROSC	170 (169, 172)	165 (160, 167)	68 (63, 71)	55 (49, 55) *	26 (13, 27)	28 (21, 42)	1.7 (1.1, 2.2)	1.2 (1.8, 4.0)
10 min post-ROSC	172 (170, 177)	145 (128, 161)	62 (61, 64)	55 (48, 58)	25 (15, 29)	35 (26, 37)	3.3 (1.9, 4.6)	2.0 (1.8, 4.0)
15 min post-ROSC	186 (174, 196)	146 (137, 152) *	61 (60, 62)	46 (37, 52) *	26 (20, 28)	28 (28, 30)	3.3 (2.3, 4.4)	4.4 (3.7, 5.0)

Values represented are median (IQR). BP = blood pressure; DO_2_ = oxygen delivery; Q_CA_ = carotid blood flow; and ROSC = return of spontaneous circulation. * *p*-value < 0.05 compared to the corresponding control value.

## Data Availability

The data presented in this study are available on request from the corresponding author due to unpublished data.

## Supplementary Materials

The following supporting information can be downloaded at: https://www.mdpi.com/article/10.3390/children13050651/s1. Table S1: Characteristics and resuscitation data only including lambs that achieved return of spontaneous circulation with an intact cord (120 s); Table S2: Hemodynamics and EPI concentration only including lambs that achieved return of spontaneous circulation with an intact cord (120 s).

## Abbreviations

The following abbreviations are used in this manuscript:

CCs	chest compressions
DCC	delayed cord clamping
EPI	epinephrine
ICC	immediate cord clamping
PPV	positive pressure ventilation
ROSC	return of spontaneous circulation
UV	umbilical vein
