# Supplementary material for: Advanced Resuscitation with an Intact Cord in Preterm Lambs: A Feasibility Trial
Source: Children (Basel). 2026 May 6;13(5):651. doi: 10.3390/children13050651 (PMC13204569; doi:10.3390/children13050651)
Supplement: Supplementary file 1 [file children-13-00651-s001.zip › children-4282998-supplementary.pdf]

Supplemental Table S1: Characteristics and Resuscitation Data Only Including Lambs that Achieved Return of Spontaneous Circulation with an Intact Cord (120s)

Groups	Control (n=5)	Intervention (n=4)
Weight (kg)	3.5 (3.0, 3.6)	2.5 (2.0, 2.9)
Gestation (days)	126 (125, 126)	125 (125, 125)
Arterial pH		
Baseline	7.23 (7.21, 7.25)	7.23 (7.21, 7.24)
Asphyxia	6.87 (6.84, 6.90)	6.88 (6.86, 6.90)
ROSC	6.78 (6.77, 6.80)	6.84 (6.83, 6.85)*
5-min post ROSC	6.71 (6.69, 6.73)	6.78 (6.76, 6.79)*
15-min post ROSC	6.75 (6.74, 6.76)	6.85 (6.75, 6.91)
Arterial PO_2 (mm Hg)		
Baseline	21 (18, 24)	19 (17, 21)
ROSC	21 (17, 23)	15 (9, 19)
5-min post ROSC	33 (32, 34)	28 (18, 35)
15-min post ROSC	74 (54, 88)	63 (44, 81)
Arterial PCO_2 (mm Hg)		
Baseline	70 (62, 70)	69 (62, 74)
Asphyxia (mm Hg)	136 (120, 136)	131 (123, 138)
ROSC (mm Hg)	159 (152, 165)	132 (123, 141)*
5-min post ROSC (mm Hg)	141 (136, 142)	121 (110, 132)
15-min post ROSC (mm Hg)	2.6 (2.1, 3.2)	3.0 (2, 3.8)
Arterial Lactate (mmol/L)		
Baseline	7.3 (7, 7.5)	7.7 (7.1, 8.1)
Asphyxia	9.5 (9.4, 9.9)	8.5 (8.0, 8.7)*
ROSC	8.7 (8.5, 9.6)	9.1 (8.5, 9.5)
5-min post ROSC	8.3 (8.0, 9.3)	8.4 (7.6, 9.2)
15-min post ROSC		
FIO_2		
At ROSC	1 (1, 1)	0.53 (0.53, 0.6)*
5-min post ROSC	0.75 (0.6, 1)	1 (1, 1)
15-min post ROSC	0.61 (0.5, 0.7)	0.77 (0.65, 1)
Time to HR < 30/min (min)	18 (11.4, 22.2)	17 (12.1, 22.6)
Time to first EPI (min)	3.7 (3.4, 5.2)	0.96 (0.8, 1.5)*
Got chest compressions (%)	5 (100%)	0
ROSC success	4 (80%)	4 (100%)
> one EPI dose	1 (20%)	0
Time to ROSC	5.7 (4.4, 5.7)	1.8 (1.5, 2.2)*

Values represented as median (IQR). HR = heart rate; ROSC = return of spontaneous circulation. *p-value < 0.05 compared to corresponding control value.

Supplemental Table S2: Hemodynamics and EPI concentration Only Including Lambs that Achieved Return of Spontaneous Circulation with an Intact Cord (120s)

Time	Heart Rate (bpm)		Mean BP (mmHg)		Left Q_{CA} (ml/kg/min)		DO_2 (ml O_2/kg/min)		EPI concentration (ng/mL)	
	Control	Intervention	Control	Intervention	Control	Intervention	Control	Intervention	Control	Intervention
Fetal baseline	129 (25)	151 (19)	51 (15)	50 (9)	32 (7)	38 (9)	2.4 (0.9)	3.5 (0.9)	0	0
Bradycardia	43 (9)	49 (8)	13 (17)	20 (3)	2.8 (4)	2.3 (1)	0.01 (0)	0.01 (0)	2.7 (2.6)	1.3 (2.6)
ROSC	182 (23)	116 (22)*	33 (8)	58 (11)*	15 (11)	31 (13)	1.1 (1.3)	1.6 (1.8)	275 (64)	283 (128)
1-min post-ROSC	197 (13)	172 (12)*	58 (16)	66 (12)	22 (8)	35 (14)	1.1 (0.3)	1.9 (1.8)	166 (25)	128 (56)
2-min post-ROSC	189 (15)	166 (8)*	59 (15)	65 (20)	21 (7)	34 (11)	2 (1.4)	1.7 (1.6)	80 (25)	72 (33)
3-min post-ROSC	180 (8)	162 (17)	67 (5)	56 (10)	21 (7)	35 (11)	2.1 (1.3)	1.7 (1.9)		
4-min post-ROSC	172 (10)	170 (24)	65 (5)	57 (8)	21 (7)	40 (12)*	2.1 (1)	2.9 (3.8)		
5-min post-ROSC	171 (15)	164 (13)	66 (7)	53 (4)*	22 (8)	41 (12)*	1.9 (0.6)	3.5 (4.0)	40 (13)	39 (20)
10-min post-ROSC	170 (13)	149 (39)	63 (5)	57 (8)	26 (10)	44 (14)*	3.8 (1.6)	5.5 (4.2)	14 (1.1)	20 (13)
15-min post-ROSC	180 (15)	153 (28)	61 (3)	46 (10)*	27 (7)	34 (6)	3.9 (1.5)	4.7 (1.2)	12 (4.6)	10 (5.5)

Values represented in mean (SD). BP = blood pressure; DO_2 = oxygen delivery; Q_{CA} = carotid blood flow; ROSC = return of spontaneous circulation. *p-value < 0.05 compared to corresponding control value.
